# Chemical & Nano-mechanical Study of Artificial Human Enamel Subsurface Lesions

**DOI:** 10.1038/s41598-018-22459-7

**Published:** 2018-03-06

**Authors:** R. Al-Obaidi, H. Salehi, A. Desoutter, L. Bonnet, P. Etienne, E. Terrer, B. Jacquot, B. Levallois, H. Tassery, F. J. G. Cuisinier

**Affiliations:** 1 LBN, Univ. Montpellier, Montpellier, France; 2 Aix Marseille University, Marseille, France; 3 Laboratoire Charles Coulomb (L2C), UMR 5221 CNRS-Université de Montpellier, Montpellier, FR-34095, France

## Abstract

White lesions represent an early phase of caries formation. 20 human sound premolars were subjected to pH cycling procedure to induce subsurface lesions (SLs) *in vitro*. In addition, 2 teeth with naturally developed white spot lesions (WSLs) were used as references. All specimens characterized by confocal Raman microscopy being used for the first time in examining white & subsurface lesions and providing a high resolution chemical and morphological map based on phosphate peak intensity alterations at 960 cm^−1^. Nanoindentation technique was used to measure Hardness (H) and Young’s modulus (E) of enamel. Phosphate map of examined samples exhibited presence of intact surface layer (ISL) followed by severe depletion in (PO_4_^3−^) peak in the area corresponding to the body of the lesion. In all examined groups, the mechanical properties of enamel were decreased in lesion area and found to be inversely related to penetration depth of indenter owing to enamel hierarchical structure. By combining the above two techniques, we linked mechanical properties of enamel to its chemical composition and ensured that the two methods are highly sensitive to detect small changes in enamel composition. Further work is required to bring these two excellent tools to clinical application to perceive carious lesions at an early stage of development.

## Introduction

Subsurface enamel demineralization is known as white spot lesions (WSLs); they represent the early stage of caries formation where affected surfaces seem to be intact upon gentle probing. However, with absence of effective treatment, cavitation may occur thereby increasing the necessity of invasive restorative treatments^1,2^.

The WSL presents itself as a milky white opacity on smooth surfaces of teeth^3^. By examining histological sections of WSL, four zones could be distinguished: relatively intact surface layer (ISL), body of the lesion, dark zone and translucent zone which represents advancing front of the lesion^4^.

The translucency of enamel is an optical phenomenon that depends on size of intercrystalline spaces. In early stages, active caries requires air drying to be visible, as the dissolution process of crystals at outer enamel surface begins. Further enlargement of intercrystalline spaces results in white patch visible without dryness. The effect of dehydration on enamel translucency is resulting from replacement of water content around enamel prisms with air. In a heterogeneous system, like enamel prisms surrounded by a fluid medium, scattering occurs due to difference in refractive indices (RI) of the two involved components. As RI of enamel is approximately 1.65, while that of water is 1.33 and of air is 1.00. Hence, larger difference in RI values, produces greater scattering at the enamel/air interface^5^.

Every day, demineralization and remineralization occur in the mouth several times as an active process, with progression or reversal of dental caries being the end result. The process of dental caries can be modeled in the laboratory to produce the early manifestation of caries, namely, the subsurface lesions (SLs). The dynamic nature of the process has been modeled in numerous laboratories by various pH cycling models^6,7^ in an attempt to simulate intraoral conditions in which enamel is subjected to repeated sequences of de/remineralization periods. The advantage of those models is that much can be learned about the processes involved, in a shorter period of time.

Transverse microradiography (TMR) is an essential method to determine the amount of mineral gain or loss in subsurface carious lesions *in vitro*. In addition, it has become a standard method, by which other recently developed caries detection techniques are compared and validated^8,9^. Though, preparing sections as thin as 100 µm that need to be very plane to ensure accuracy of the measurements; representing a major problem for this method. This fact makes this technique destructive and time consuming. Particularly, in case of enamel, probably because of its brittleness, this method was frequently found to yield irregular sections^10^.

Per contra, Raman microscopy which is a non-invasive technique; requires minimal specimen preparation, less expensive in term of cost and allows for simultaneous characterization of organic and inorganic tooth phases^11^. The high spatial resolution (300 nm) makes it an excellent tool for analyzing human enamel components, enabling the detection of WSL at an early stage of development^12^. Lesion depth measurements obtained from Raman scans; are based on phosphate peak intensity alterations which are closely related to its content in a given section^13^. This fact is even making the definition of the outer surface of a lesion easier when it is difficult to delineate it under TMR^14^.

Nanoindentation (NI) technique enables investigations of local mechanical properties under various loading regimes based on load displacement data of indentations on submicron scale. Using this technique to measure mechanical characteristics at multiple locations within the same enamel sample is appropriate because it can accurately measure the mechanical properties of very small volumes with fine spatial resolution and show high sensitivity to any change affecting their values^15,16^.

Our approach of combining confocal Raman microscopy, being used for the first time in examining white and subsurface lesions, with nanoindentation technique will add chemico-mechanical specificity in providing important information about an artificial model of subsurface lesions in human teeth to facilitate the future investigations on the efficacy WSLs treatments.

## Materials and Methods

### pH cycling procedure

(2) Premolars with naturally developed WSL (used as references for comparison purposes), in addition to (20) sound premolars (without any enamel developmental defects), were employed in this study. These teeth were extracted for orthodontic reasons and were used after obtaining approval of the local ethical research committee (process No. 2017–2907) and informed consent from all subjects. All procedures were carried out in accordance with relevant guidelines and regulations.

The number of teeth required in this study was calculated by using BiostaTGV site^17^. By comparing two means that were observed during our preliminary studies; we found that the minimum number of teeth in each group is 5 teeth per group.

Teeth washed with de-ionized water to remove any debris, stored in de-ionized water with 0.1% antimicrobial thymol and kept in refrigerator at 4 °C until use. Teeth were polished with non-fluoridated paste and further cleaned ultrasonically^18^.

To create enamel subsurface lesions; 20 sound teeth were randomly divided into 4 groups which undergone 5, 6, 7 and 8 pH cycles respectively. Each cycle lasted 24 h. Different numbers of cycles were used in order to produce SLs that resemble, as much as possible, the naturally developed white lesions which classified as score 1 and 2 according to ICDAS^19^. Teeth were subjected to pH cycling procedure; using a demineralizing solution (0.075 M/L acetic acid, 1.0 mM/L calcium chloride and 2.0 mM/L potassium phosphate, pH = 4.3) and a remineralizing solution (150 mM/L potassium chloride, 1.5 mM/L calcium nitrate, 0.9 mM/L potassium phosphate, pH = 7). All chemicals were supplied by Sigma-Aldrich, France.

The procedure includes the immersion of each tooth in 20 ml of demineralizing solution for 6 hours at 37 °C. Then it removed and rinsed with distilled water for 2 min. before immersion in 20 ml of remineralizing solution for 18 hours at 37 °C. Adhesive tape disk of 6 mm diameter was cut and burnished on the buccal surface of each tooth. An acid resistant nail varnish was used to cover the whole surface of each tooth; the tape was removed leaving a window of 6 mm in diameter on buccal surface.

After the achievement of different number of cycles; teeth were divided longitudinally into two halves by using high speed diamond saw (Isomet 2000, Buehler, USA) to produce cross sections of teeth which then embedded in self-curing acrylic resin and keeping the cross sectioned surfaces exposed. The sections were polished by SiC papers #800, #1000 and #1200, followed by diamond pastes, using a polishing machine (Escil, France).

### Raman microscopy

Raman spectra are recorded using a Witec Confocal Raman Microscope System alpha 300 R (Witec Inc., Ulm, Germany). Excitation in the confocal Raman microscopy is assured by a frequency doubled Nd: YAG laser (Newport, Evry, France) at a wavelength of 532 nm. The incident laser beam is focused onto the sample through a ×20 NIKON objective (Nikon, Tokyo, Japan). Then Raman backscattered radiation mixed with Rayleigh scattered are passed through an edge filter to block the Rayleigh’s. Acquisition time of a single spectrum was set to 0.05 s. Each spectrum corresponding to a spatial unit defined as a voxel (300 *nm *× 300 *nm *× 1 μ*m*). All data acquisition and processing were performed using Image Plus software from Witec.

Chemical mapping of dental enamel was carried out over cross sections of all specimens with naturally and artificially developed subsurface lesions. The phosphate (PO_4_^3−^) ion has four internal vibrational modes. We chose to observe changes in intensity of the strongest peak at 960 cm^−1^ which is attributed to *v*_1_ (PO_4_^3−^) symmetric stretching mode.

Using an indicative look-up table (LUT), red hues indicate highest phosphate intensities, CO_3_^2−^/PO4^3−^ ratio and crystallinity rates while purple hues represent lowest value of previous variables in chosen region. K-mean cluster analysis (KMCA) divides data into K mutually exclusive clusters which are compact and well-separated. Each cluster represents a zone depending upon magnitude of its phosphate peak intensity. Four zones with pseudo color were distinguished^11^.

Crystallinity degree in enamel is determined by measuring changes in peaks ratio of symmetric mode of phosphate at 960 cm^−1^ over 950 cm^−1^ ^20^.

Raman peak at 1070 cm^−1^ is assigned to B-type carbonated hydroxyapatite (CHA).The ratio of intensities of carbonate to phosphate at (1070/960 cm^−1^) were calculated throughout the whole section of each sample in order to detect variations in carbonate content in different zones of each sample^21^.

### Nanoindentation technique

A nanoindenter equipped with a Berkovich tip (CSM, Switzerland), was used to measure hardness (H) and Young’s modulus (E) of enamel. Tests were made in lesion, intermediate and sound enamel areas respectively. Indentations with a maximum load of 10 mN were marked on the samples where nanoindentation test was performed. The distance between each 2 neighboring indentations was at least 10 μm. A load of 20 μN was set for all tests, and loading and unloading time was 270 s. At least; ten indentations were done on each zone of the examined enamel surface.

H and E variables of samples were obtained from load-displacement loading/unloading curves, using Oliver and Pharr method^22^, which is the most successful model for nanoindentation data analysis.

The hardness (H) is calculated at the maximum force according to the following relation:

1 $${\rm{H}}=\frac{{\rm{F}}}{{{\rm{A}}}_{{\rm{P}}}}$$

where F is the maximum force and A_p_ is the projected surface contact area between the indenter and the sample.

The Young’s modulus (E) of the sample can be obtained using the slope (S) at the beginning of the unloading curve according to the following relations:

2 $${E}^{\ast }=\frac{\sqrt{{\rm{\pi }}}}{2\beta }\frac{{\rm{S}}}{{\sqrt{{\rm{A}}}}_{{\rm{P}}}}$$

and

3 $$\frac{1}{{E}^{\ast }}=\frac{1-{\nu^{2}}}{E}+\frac{1-{\nu }_{i}^{2}}{{E}_{i}}$$

where β is a geometrical constant depending upon indenter shape (1.034 in our case), E_i_ (1070 GPa) and ν_I_ (0.07) are Young’s modulus and Poisson’s coefficient of diamond indenter respectively. ν is the Poisson’s coefficient of the sample which equals to 0.3 for enamel^23^.

Overall mean and standard deviation (SD) for lesion depth (measured from Raman’s data), penetration depth (Pd), H and E of different groups (from nanoindentation test) were calculated. Statistical analysis was performed using One Way ANOVA Analysis of Variance for all experimental groups, followed by multiple comparison procedures between each two pairs with Holm-Sidak method. All statistical procedures were performed at over all significant level of α = 0.05 with SigmaPlot version 11.0 (Systat Software, Inc., USA).

## Results

**Figure 1:**
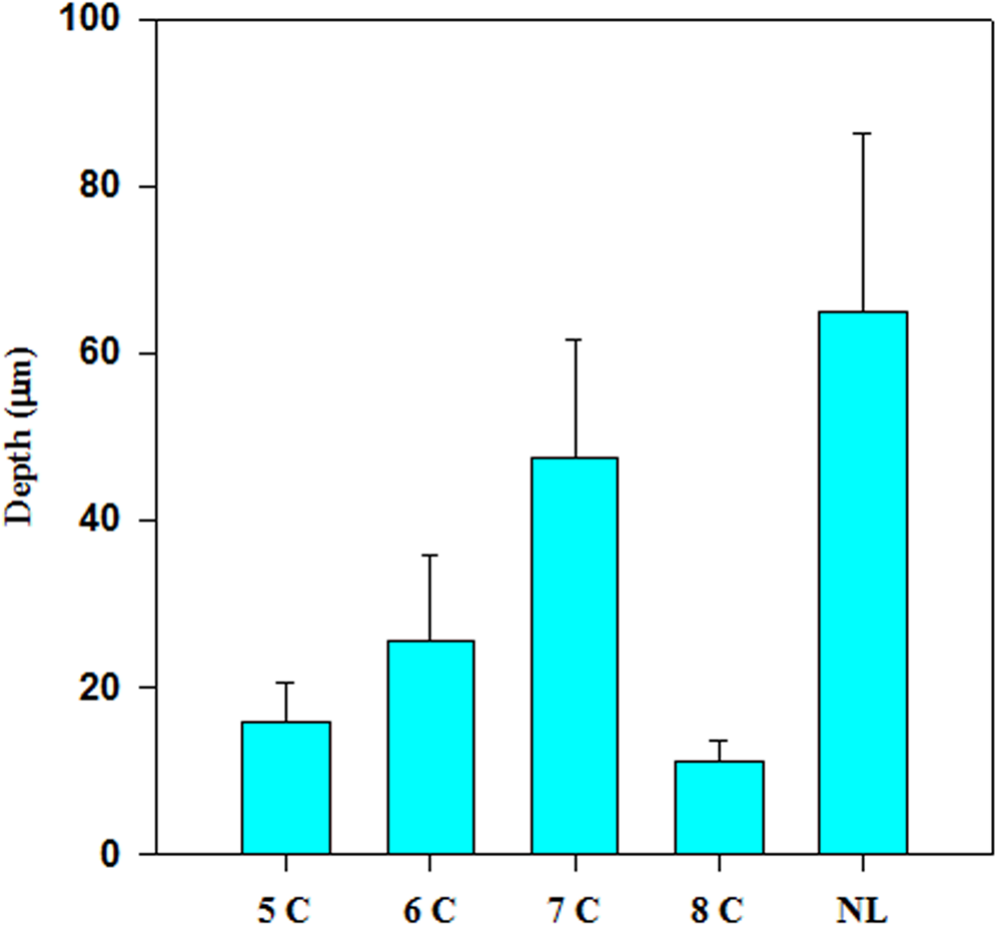
The relation between means of lesion depth determined by measuring variations in PO_4_^3−^ peak intensity at 960 cm^−1^ & number of cycles (C). SD values are represented by error bars. (NL) natural lesion.

Depth of artificial subsurface lesions was measured and compared to that of natural lesion. Depth measurements were based on phosphate peak intensity alterations and found to increase linearly with gradual rise in number of cycles except for 8 cycles lesion, where a considerable loss of enamel layer has taken place. A statistically significant difference (p < 0.05) was found between all examined groups (Fig. 1).

**Figure 2:**
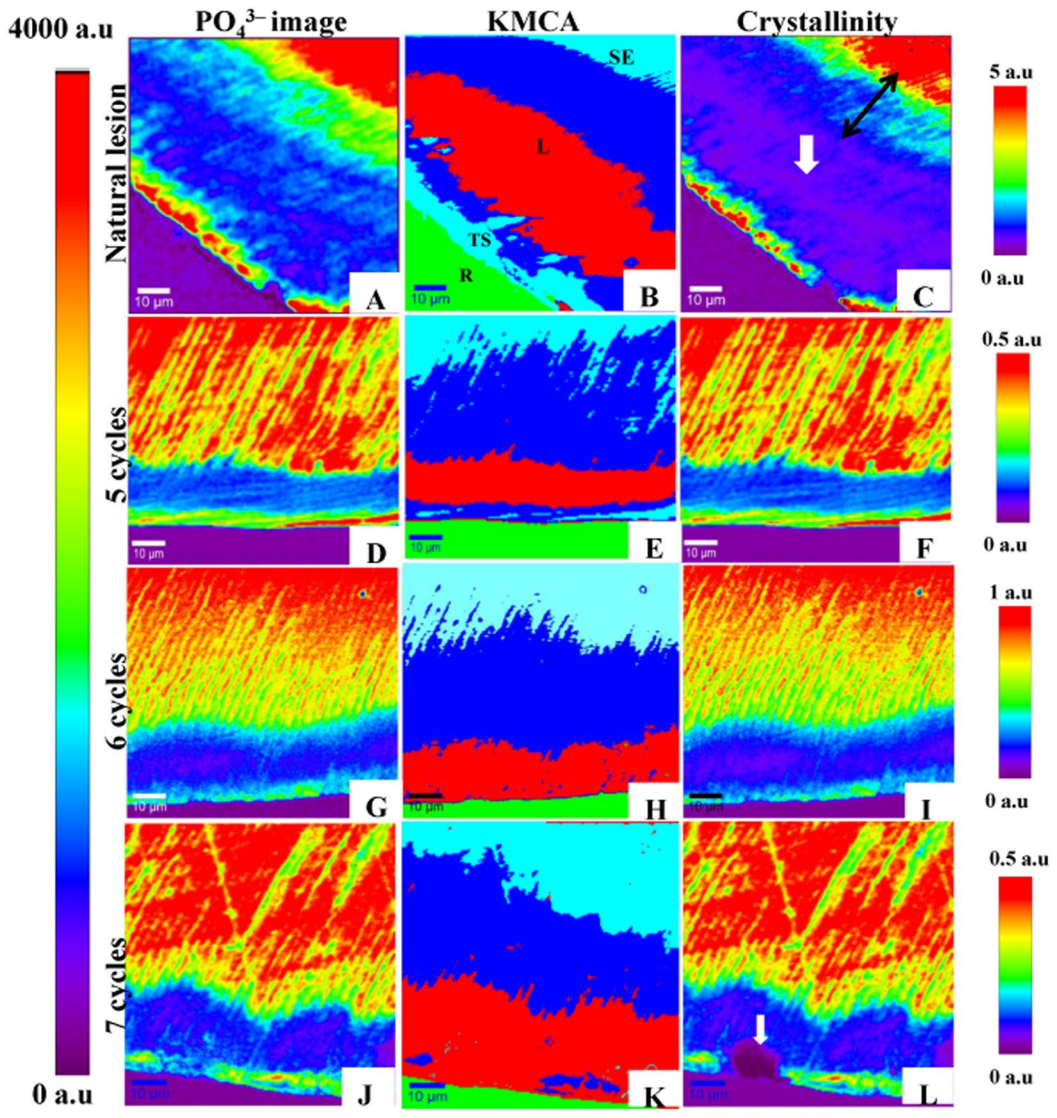
Natural lesion versus *in-vitro* lesions exposed to 5, 6 and 7 cycles. (**A**,**D**,**G** and **J**) images constructed from phosphate peak intensity at 960 cm^−1^. Look Up Table (LUT) on the left. (**B**,**E**,**H** and **K**) K-mean cluster analysis (KMCA) images, green zones are corresponding to embedding resin. (**C**,**F**,**I**, and **L**) images of crystallinity (phosphate peaks ratio at (960) over (950) cm^−1^). Individual LUT on the right. Purple zones are corresponding to embedding resin. Crystallinity images are almost following phosphate pattern in the 1^st^ group of images, except for C and L images; where we can observe the presence of purple hues (white solid arrows) in lesion area which indicate total loss of crystallinity in lesion area. (Following symbols in image (**B**) are valid for all images: SE; sound enamel, L; lesion, TS; tooth surface, R; resin).

Raman acquisitions were carried out from outer enamel surface towards dentin-enamel junction (DEJ). Images constructed from phosphate peak intensity at 960 cm^−1^ are shown in (Fig. 2A,D,G,J). All examined samples exhibited phosphate signal at outer enamel surface which indicates the presence of ISL. A severe depletion in (PO_4_^3−^) peak in the area corresponding to body of the lesion. At greater distances into the enamel; phosphate peak intensity converges to that of sound enamel signaling the end of the lesion.

The reconstructed images of enamel crystallinity of all groups, almost exhibit a pattern similar to that of PO_4_^3−^ images (Fig. 2C,F,I,L). Each pseudo color in these images; represents a special crystallinity value. Leveled color scale bars are designed to estimate the value of each color. Crystallinity decreases abruptly in lesion zone (blue). Thereafter; it starts to increase gradually in the intermediate zone (green, yellow hues) before it reaches to its maximum value in sound enamel (red area) beyond subsurface lesion.

Results of K-mean cluster analysis of data set of four clusters are shown in (Fig. 2B,E,H,K). Images constructed via KMCA demonstrating clusters with four distinguished colors: acrylic resin (green), sound enamel with two different intensities of phosphate (in turquoise zone, PO_4_^3−^ intensity is stronger than that in blue zone) and demineralized lesion body (red).

**Figure 3:**
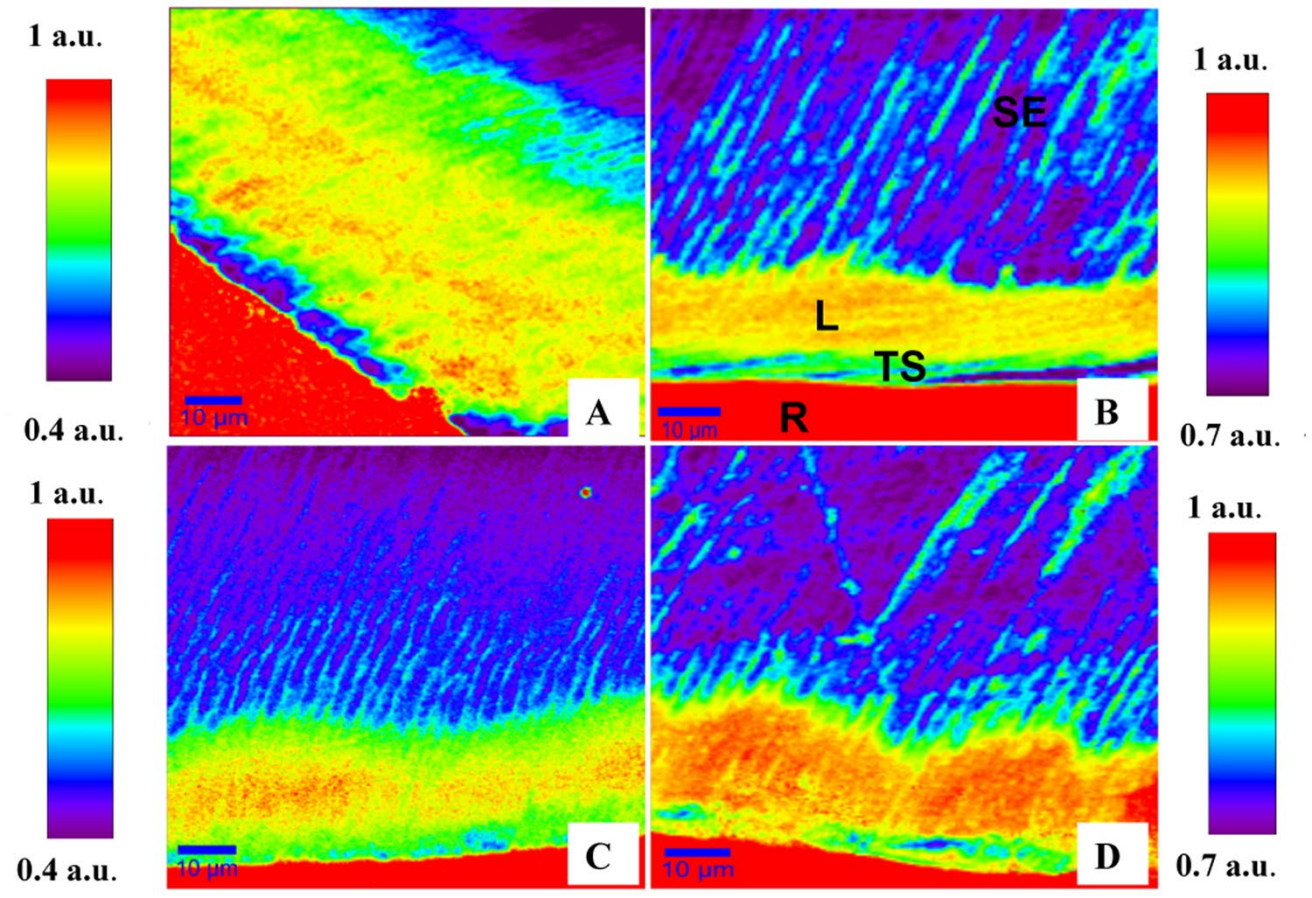
(**A**) Natural lesion versus *in-vitro* lesions (**B**,**C**,**D**) exposed to 5, 6 and 7 cycles respectively. Images constructed from CO_3_^2−^/PO_4_^3−^ ratio at (1070 cm^−1^) over (960 cm^−1^). Individual LUT on left & right where red hues indicate highest CO_3_^2−^/PO_4_^3−^ ratio and purple hues represent lowest values of the same ratio. (Following symbols in image B are valid for all images: SE; sound enamel, L; lesion, TS; tooth surface, R; resin).

Reconstructed images derived from CO_3_^2−^/PO4^3−^ ratio (Fig. 3A–D) were used to analyze changes in enamel inorganic components in each zone of the lesion. They revealed an increase in CO_3_^2−^/PO4^3−^ ratio in lesion zone in comparison to sound enamel zone.

**Figure 4:**
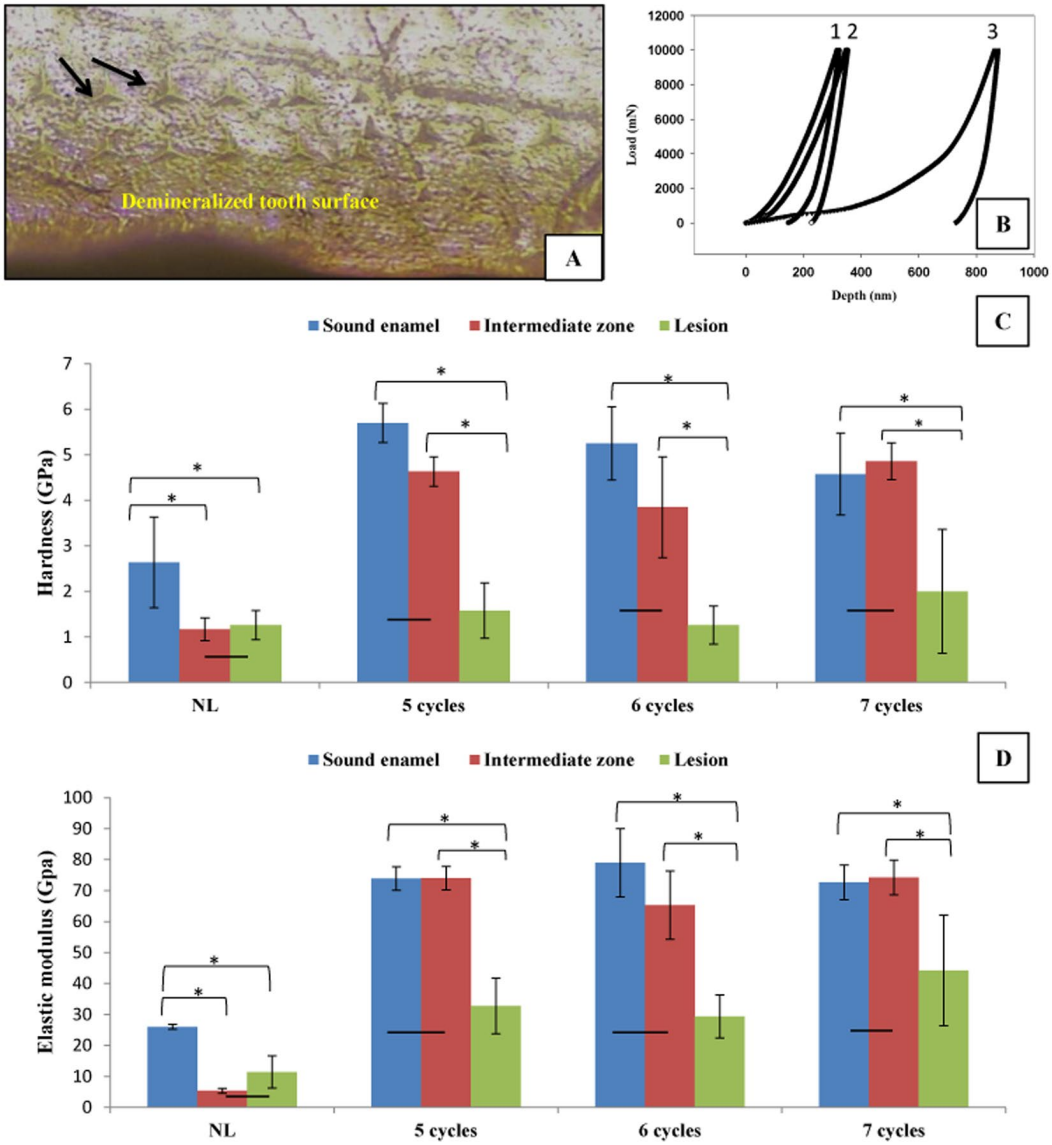
(**A**) Optical microscopic image of polished enamel surface with prints of Berkovich tip indenter in lesion zone. (**B**) A graph demonstrating the nanoindentation load-displacement curves in: 1 sound enamel, 2 intermediate zone & 3 lesion area. The difference in penetration depth (Pd) between the three zones is very obvious; indicating an inverse relationship between the reduction in hardness value and the increase in the depth of penetration. (**C** and **D**) bar graphs showing H and E mean values in all zones in: Natural lesion versus artificial lesions after 5, 6 and 7 cycles consecutively. Asterisk (*) shows a statistically significant difference (p < 0.05) between zones connected by bracket within each group. Horizontal bar indicates no significant difference (p > 0.05) among subgroups marked by it.

Nanoindentation load-displacement curve is drawn from applied load versus depth profile (Fig. 4B). Curves were derived from different indentations made in three zones of enamel: unaffected enamel close to dentin, lesion area which is confined to enamel surface and intermediate zone which lies between the two areas to show differences in penetration depth of indenter tip.

H and E of enamel were determined in all zones of each group to measure intra-tooth variations. A reduction in two variables value was detected in lesion area in all examined groups (Fig. 4C,D). E rates display a positive relationship with those of H, except for the natural lesion (NL) where E value is smaller than expected. Difference was found to be significant (p < 0.05) by comparing E value of (NL) with those of other artificial lesions. While, the difference was found to be non-significant (p > 0.05) by comparing changes in rates of Pd and H of lesions (Table 1).

**Table 1:** Penetration depth (Pd), Hardness (H), elastic modulus (E) in lesion area of natural white spot lesion (N = 2) & artificial lesions of 5, 6 & 7 cycles (N = 5 in each group).

Tested area	Pd (µm) Mean ± SD	H (GPa) Mean ± SD	E (GPa) Mean ± SD
SE	0.3 ± 0.01	4.5 ± 0.67	74.7 ± 3.3
NWSL	0.54 ± 0.09	1.26 ± 0.32	11.4 ± 5.2
5 cycles lesion	0.5 ± 0.08	1.58 ± 0.61	32.8 ± 9
6 cycles lesion	0.6 ± 0.24	1.1 ± 0.52	27.5 ± 8.2
7 cycles lesion	0.5 ± 0.2	2 ± 1.4	42.5 ± 18.4

Difference was found to be significant (p < 0.05 between E value of NL (natural lesion) & those of other artificial lesions, while non-significant difference (p > 0.05) was found between Pd and H values of all kinds of lesions.

## Discussion

Chemical composition and mechanical properties of WSLs and subsurface artificial lesions were described using Raman microscopy and nanoindentation techniques. Both techniques tested the same areas of each sample.

To our knowledge, confocal Raman microscopy has been used for the first time in this study to detect WSLs and experimentally induced subsurface lesions. It can provide a high resolution chemical and morphological map of examined specimen, detecting even very small changes in its chemical composition. Data analysis of each acquired scan which is comprised of tenth thousands of single spectrums, is used to reconstruct different Raman detailed images. Transverse microradiography is a quantitative method depending on x-ray absorbance of an object and images reflect mineral concentration in mineralized tissue slices^24^. Systematic and random errors represent the two sources of errors that could affect x-ray absorbance measurement. The crucial factor with regard to systematic errors is the beam inhomogeneity, while, film choice is the vital factor in respect to random errors^24^. Moreover, artifacts result from inadequate section preparation as we mentioned before, may incorrectly be interpreted as additional demineralization in the lesion part of the specimen. Furthermore, TMR is usually applied, to determine overall changes in mineral content and not detailed structures of biological tissues^25^. TMR resolution is dependent on X-ray detector resolution, i.e., silver grain diameter of radiographic film and on the microscope scanner used to read the film. High resolution microradiograph is with a pixel resolution of 2.15 µm^26^, comparing to a voxel size of (300 × *nm* × 300 *nm* × 1 *μm* for confocal Raman microscopy. Hence, Raman microscopy could be considered as a superior alternative for Raman spectroscopy and transverse microradiography^12^.

Deepest subsurface lesion that we could produce *in vitro* is shallower than naturally developed one, in spite of lower concentrations of calcium, phosphate, and even lower pH values that have been used to induce a faster demineralization *in vitro*. Besides that, thickness of subsurface lesions reported by other studies^27,28^ was greater than thickness of this study. However, there is less detailed information about these fabricated lesions, in particular, the evidences concerning the presence of ISL.

Enamel is composed of large number of hydroxyapatite crystals (HACs); represented by the chemical formula Ca_10_(PO4)_6_(OH)_2_ with incorporation of many cations and anions in their lattice resulting in the formation of different kinds of apatite. The main substituent in biological apatite is carbonate ion (CO_3_^2−^) which substitutes the phosphate group (PO_4_^3−^) to form B-type CHA containing 4–6 wt % carbonate^29,30^. Incorporation of carbonate into enamel increases its dissolution rate by interfering with crystal structure^31^. In particular, crystallinity, i.e., crystal size and perfection of apatite are of great importance, because small, more imperfect crystals due to presence of substituents, make them more susceptible to acid dissolution and caries progression^32^. Therefore, lowering in enamel crystallinity in lesion zone of our samples could be related to the increase in CO_3_^2−^/PO_4_^3−^ ratio in the same zone^33–35^.

KMCA method assembles set of spectra of the same or close intensities in a well-recognized cluster^11^. High resolution images constructed by KMCA visibly reveal changes in enamel structure associated with number of cycles. As ISL becomes thinner, less mineralized and more porous; thickness of demineralized area increases progressively with time, until the eighth cycle, where enamel surface dissolved. These results emphasize the fact that degree of enamel dissolution strengthens with repeated and prolonged variations in pH values.

Mechanical properties of enamel are inconstant and dependent on its chemical composition and structural organization. Enamel is anisotropic material where prisms are arranged in pattern perpendicular to their long axis, so that any variations in its prisms direction and composition may lead to differences in its mechanical properties^36^.

Our results showed no significant difference between mechanical properties of intermediate zone and lesion area of NL (Fig. 4C,D) contrary to other artificial lesions. Simultaneously, we exclusively observed that crystallinity degree has obviously declined in intermediate zone of NL (Fig. 2C, double ended black arrow) which indicates an alteration in crystal size and shape and consequently reduced apatite perfection which constitutes the structural unit of enamel prisms. These modifications in enamel structure could help in explaining the reduced mechanical properties of intermediate zone of WSL.

Outer enamel layers are harder than inner layers which are close to DEJ, where enamel becomes less compact due to increase inter-prismatic voids and less mineralized due to decrease mineral density and increase organic matrix contents. This could explain the low H and E values for sound enamel in NWSL group (Fig. 4C,D), where indentations in this zone were made next to DEJ because of extensive depth of NWSL (>60 µm)^37,38^.

Table 1 demonstrates how the mechanical properties of enamel (hardness and elastic modulus) are inversely related to penetration depth of indenter owing to enamel hierarchical structure. Obtained average values of H ~ 4.5 GPa and E ~ 74.7 GPa for sound enamel at 10 mN load are consistent with values reported in previous study where E ~ 80 GPa and H ~ 4 GPa^39^.

Mechanical properties were considerably reduced in demineralized enamel compared to sound enamel as a result of inorganic substance loss (Table 1). Records obtained from the above two methods show similar changes across the lesion and represent a mechanism for linking mechanical properties of enamel to its composition which is in coincident with prior studies^35,40^.

We can conclude that our protocol is reliable to reproduce subsurface lesions *in vitro* in a relatively short period as long as it is limited to seven cycles to ensure the presence of highly mineralized ISL which represents the characteristic feature of these lesions which complicates their clinical non-invasive treatment. These artificial models with methods of characterization, particularly confocal Raman microscopy, are typical to test the efficacy of remineralizing dental products. Our work certifies that the two methods are highly sensitive to detect small changes in enamel composition. Further work is required to facilitate their use in the dental clinic to detect carious lesions at an early stage of formation.
